# Spherical Oligo-Silicic Acid SOSA Disclosed as Possible Endogenous Digitalis-Like Factor

**DOI:** 10.3389/fendo.2014.00233

**Published:** 2015-01-23

**Authors:** Franz Kerek, Victor A. Voicu

**Affiliations:** ^1^SiNatur GmbH, Martinsried, Germany; ^2^Department of Clinical Pharmacology, Toxicology and Psychopharmacology, Carol Davila University of Medicine and Pharmacy, Bucharest, Romania

**Keywords:** oligo silicic-acid, ATPase regulation, digitalis-like factor, ouabain

## Abstract

The Na^+^/K^+^-ATPase is a membrane ion-transporter protein, specifically inhibited by digitalis glycosides used in cardiac therapy. The existence in mammals of some endogenous digitalis-like factors (EDLFs) as presumed ATPase ligands is generally accepted. But the chemical structure of these factors remained elusive because no weighable amounts of pure EDLFs have been isolated. Recent high-resolution crystal structure data of Na^+^/K^+^-ATPase have located the hydrophobic binding pocket of the steroid glycoside ouabain. It remained uncertain if the EDLF are targeting this steroid-receptor or another specific binding site(s). Our recently disclosed spherical oligo-silicic acids (SOSA) fulfill the main criteria to be identified with the presumed EDL factors. SOSA was found as a very potent inhibitor of the Na^+^/K^+^-ATPase, Ca^2+^-ATPase, H^+^/K^+^-ATPase, and of K-dp-ATPase, with IC_50_ values between 0.2 and 0.5 μg/mL. These findings are even more astonishing while so far, neither monosilicic acid nor its poly-condensed forms have been remarked biologically active. With the diameter ϕ between 1 and 3 nm, SOSA still belong to molecular species definitely smaller than silica nano-particles with ϕ > 5 nm. In SOSA molecules, almost all Si-OH bonds are displayed on the external shell, which facilitates the binding to hydrophilic ATPase domains. SOSA is stable for long term in solution but is sensitive to freeze-drying, which could explain the failure of countless attempts to isolate pure EDLF. There is a strong resemblance between SOSA and vanadates, the previously known general inhibitors of P-type ATPases. SOSA may be generated endogenously by spherical oligomerization of the ubiquitously present monosilicic acid in animal fluids. The structure of SOSA is sensitive to the concentration of Na^+^, K^+^, Ca^2+^, Mg^2+^, and other ions suggesting a presumably archaic mechanism for the regulation of the ATPase pumps.

## Introduction

Investigating the staircase effect on frog ventricular muscle, it was revealed in the early 1950s that human serum contains a factor, which improves the contractile power of the heart, similar to plant-derived digitalis glycosides (1). Besides the serum, this digitalis-like activity was reproduced by a total extract of adrenal but, individual cortisol steroids were, with one exception, inactive. Referring to their finding Szent-Györgyi concluded (2) in more general terms that “such digitalis-like factors are ubiquitously distributed in mammals” and “digitalis glycosides from plants are actually not drugs but only substitutes of the body’s own digitalis-like factors.” This kind of relationship between exogenous drug-substances, which fits accidentally into the receptor of a body’s own factor has been verified by the discovery of endorphins, the endogenous counterparts of the plant-derived morphine (3). Why this broadly accepted rationale was not consequently applied in the differentiation between endogenous digitalis-like factors (EDLFs) and exogenous digitalis glycosides is a subject of the present review.

A further milestone finding of the 1950s was that digitalis glycosides inhibit the transport of Na^+^ and K^+^ ions across the erythrocyte membrane (4) and above all the seminal discovery of the membrane transport protein of the Na^+^ and K^+^-ions by Skou (5). This Na^+^/K^+^-ATPase (adenosine-triphosphatase) abbreviated NKA or sodium pump is present in the membrane of all eukaryotic cells. Every pumping cycle of the NKA moves 3 Na^+^ ions outward and 2 K^+^ ions inward, powered by the energy of a phosphate bond from ATP.

At the beginning of the 1960s, the existence of a circulating natriuretic factor was postulated by De Wardener et al. (6) showing that the blood, transfused from a saline-loaded, hypertensive dog, produces natriuresis in the recipient normotensive animal. It was further observed that the plasma ultra-filtrate of saline-loaded dogs inhibits the sodium transport in toad bladder and the volume expansion was accompanied by increasing concentrations of a sodium pump-inhibitory factor (7). Ascertaining that both effects were caused by the same factor the name natriuretic hormone was proposed. The natriuretic fraction extracted from the plasma of volume-expanded dogs inhibited the ouabain-insensitive NKA from rat kidney (8).

After the identity of the natriuretic hormone with the sodium pump-inhibitory factor was confirmed, the name EDLF came into use (9). The initial idea that EDLF could be a natriuretic peptide was rejected after disclosure of the atrial natriuretic peptide (ANP), which has, contrary to EDLF no NKA-inhibitory activity (10). Though the structure of EDLF remained obscure, some of its particular characteristics were established as for instance: its non-peptide nature or its specific interaction with different ATPase isoforms. But the failure of all attempts to obtain weighable amounts of pure EDLF impeded for more than five decades the disclosure of its chemical structure.

Na^+^/K^+^-ATPase controls a broad spectrum of essential cellular functions such as ion homeostasis, membrane potential, pH, temperature, and water osmosis, thereby regulating important physiological processes, e.g., muscle contraction, nervous signal transmission, renal sodium retention, and vascular tone. Study data in animal models and clinical observations in human beings suggest that cardiac insufficiency, essential hypertension, and other diseases may be caused by or connected to malfunction or dysregulation of the sodium pump.

The extensive research work related to the structure, characterization, mechanism of action, and physiological implications of the Na^+^/K^+^-ATPase was comprehensively reviewed by Gadsby et al. (11), Glynn (12), Kaplan (13), and Jørgensen et al. (14). Similarly, extensive reviews on the whole P-type-ATPase field have been published by Møller et al. (15), Kühlbrandt (16) and by the original contributions of Axelsen and Palmgren (17, 18) with special focus on evolutionary aspects of the P-type ATPases.

## ATPase Receptor Site

P-type ATPase is the generic designation of several ATP-driven transmembrane ion pumps found in bacteria, archaea, and eukaryotes. The prefix P refers to the ability of these proteins for phosphorylation and de-phosphorylation of their catalytic aspartate residue (15). By the binding and removal of the phosphate group, ATPases interconvert between two conformations, denoted by E1 and E2, each with different affinity to the nucleotide ATP (adenosine triphosphate) and the transported ions (16). A common feature of P-type ATPases is their inhibition by vanadate ions at micro- and sub-micro-molar concentrations.

From about 200 members of the P-type ATPase family, the most prominent pumps are the cell membrane Na^+^/K^+^-ATPase (NKA); the Ca**^2+^**-ATPase (SERCA) from sarcoplasmic reticulum (SR), the gastric H^+^/K^+^-ATPase, and the bacterial K-dp-ATPase. Based on 80–90% similarities of the amino acid (AA) sequences in the conserved regions, it is assumed that P-type ATPases evolved from a common ancestor, probably 3500 million years ago (17, 18).

A seminal breakthrough for the detailed structural understanding of the transmembrane ion pumping was achieved by the first high-resolution (2.6Å) crystal structure of the SERCA Ca^2+^-ATPase protein from SR solved by Toyoshima et al. (19). This study established the detailed 3D structure and accomplished the functional characterization of the cytoplasmic subunits designated P (phosphorylation), N (nucleotide binding), and A (actuator) domains. It was found that the ion-binding sites are surrounded by the M4–M6 and M8 transmembrane helices where M4 and M6 provide the efficient geometry for the coordination of the Ca**^2+^** ions. The over 50 Å distance between the membrane site of the Ca**^2+^**ion translocation and the cytoplasmic phosphorylation site is remarkably long.

In the next high-resolution (3.1 Å) crystal structure of the SERCA pump, the Toyoshima group applied the sesquiterpene lactone thapsigargin, to stabilize the Ca-free E2-(TG) state (20). The comparison of both crystallized forms Ca**^2+^**E1 and E2-(TG) revealed further details of the ion transport mechanism. In a following contribution (21), the structure of the Ca**^2+^**-ATPase was solved in the E1 state fixed by the ATP-analog AMPPCP. In the same year, the structure at 2.3 Å resolution of SERCA with phosphate analogs such as [MgF_4_]**^2-^** has been resolved (22). The studies of the Ca-pump fixed with ATP- or phosphate analogs have completed the structural insight into almost all important states of the pump turnover.

Resolving the crystal structure of the SERCA pump with the phosphate mimic [BeF_3_]**^-^**, Olesen et al. (23) provided support for the presence of an open ion pathway in the pump in which the transmembrane domains form a funnel-shaped geometry. As described later, the crystal structure data confirmed that P-type ATPases share the same architecture regardless of the size, charge, and number of ions that they transport. It seems that the differences are largely confined to the ion-binding pocket (23, 24).

Na^+^/K^+^-ATPase is sensitive to inhibition by digitalis glycosides (e.g., digoxin, ouabain), isolated from medicinal plants used over centuries to treat congestive heart failure. The term “digitalis” designates the entire group of cardiac glycosides and aglycones without regard of their structure and origin (25). Responsible for the cardiac activity is the aglycone, i.e., the steroid nucleus, which results after the sugar moiety from position 3 is removed. Steroid aglycones with five-membered lactone ring in position 17 are named cardenolides while those with six-membered lactone ring bufadienolides. Both terms, cardiotonic (CTS) or cardiac steroids (CSs), are in use but the adjective “tonic” is less rigorous as it refers to a species-dependent physiological response; thus, the designation CS should be preferred over CTS.

Since Na^+^/K^+^-ATPase is the target of digitalis drugs in heart failure patients, it was of prior importance to establish structure–activity relationships. A general correlation between binding affinity and NKA-inhibitory potency of cardiac glycosides was revealed (26) but some notable exceptions were also identified. By replacement of the five-membered lactone ring in cardenolides with the 2-pyrone ring of bufadienolides, the binding affinity declines but the inhibitory potency increased. Furthermore, while the removal of ouabain’s rhamnose moiety had little effect on inhibitory potency, it caused a decline in ligand binding affinity.

In a recent study, 30 different cardiac glycosides were investigated for their interaction with the α1, α2, and α3 isoforms of the human NKA expressed in *Pichia pastoris* (27). The study revealed significant isoform selectivity by digoxin glycosides but ouabain was found moderately α2 selective. The observed influence of the sugar moiety on the selectivity was surprising since according to pharmacological data this part influences rather the bioavailability and metabolism of the digitalis drugs.

Biophysical methods provided further insight into the charge transfer processes during the pump cycle. The binding of ouabain is associated with movement of electrical charges; thus, it can be followed by the charge-sensitive fluorescence indicator RH421. These data revealed that the binding of ouabain or generally of a cardiac glycoside to the Na^+^/K^+^-ATPase protein will stabilize under physiological conditions the E2P·2Na^+^ stage (28).

Na^+^/K^+^-ATPase is expressed in all animal cells and shows highly conserved AA sequences in the main α subunit (~1000 AA residues) responsible for the catalytic function, similar to that found in SERCA. This catalytic subunit of NKA has 10 transmembrane TM helices numbered M1–M10 from the amino-terminal. Experiments with punctual mutations of AA residues evidenced CS binding sequences in the extracellular loops L1/2, L5/6, and L7/L8. The cytosolic loop L2/3 contains the nucleotide (ATP) binding N site and the phosphorylation site P, while the NH**_2_** terminal and the L2/3 loop are responsible for the de-phosphorylation step. NKA contains further the heavily glycosylated ß-subunit with ca. ~300 AA and the tissue specific auxiliary γ subunits FXYD of ca. 70–180 residues The multiple regulatory potential of the NKA is explained by the existence of tissue-specific assemblies of different structural subunits or isoforms (29).

In comparison with more than 20 X-ray structures for Ca^2+^-ATPase, for NKA only 5 crystal structures at better than 5 Å resolution have been published. Some difficulties come from the source of NKA protein, which is limited to rabbit kidney and shark rectal gland, expressing selectively the α1 isoform. The lack of very high-affinity inhibitors able to fix the enzyme in one particular conformation posed further limitations to NKA crystallization (29).

The crystal structure of the pig-renal Na^+^/K^+^-ATPase with 2 Rubidium ions as K ion congeners was solved at 3.6 Å resolution by the PUMPKIN group in Denmark (30). The structure shows that the conformation of the: α unit (in Rb/K occlusion) closely matches the conformation of the Ca^2+^ ion bound state of SERCA.

The crystal structure of the Na^+^/K^+^-ATPase from shark enzyme was resolved (31) at 2.4 Å resolution in the: E2·2K~Pi state in which the pump has a high affinity to K^+^ ions. The coordination of K^+^ ions in the transmembrane sites and the critical role of the β subunit in binding of the K^+^ ions were remarked. Despite identical coordinating residues, small differences with the Ca^2+^-ATPase pump were noted.

**Figure 1:**
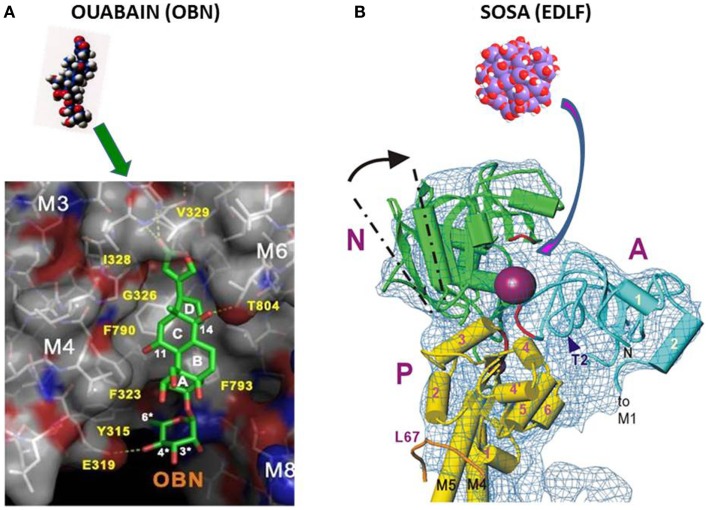
**(A)** Binding of ouabain to the hydrophobic site in NKA (32). **(B)** Binding of SOSA to the hydrophilic receptor site of decavanadate in SERCA (19).

Solving with 2.8 Å resolution, the crystal structure of Na^+^/K^+^-ATPase, co-crystallized with ouabain in the E2·2K^+^~Pi state, the authors concluded (32) that ouabain is deeply inserted into the transmembrane domain, with lactone ring near to the K^+^ binding site with partial unwinding of the M4E helix (see Figure 1A below). This unwinding should explain why ouabain binding is so slow. The data suggest reconsideration of previous data that CSs bind to the extracellular surface of the ATPase α-subunit. Since ouabain interacts with transmembrane segments M3, M4, and M6 involved in ion transport this steroid can influence or block these processes.

Based on the crystal structure at 4.6 Å resolution of the pig kidney Na^+^/K^+^-ATPase with ouabain bound in the E2P state, it was suggested that the high-affinity binding of ouabain stabilizes the phosphorylated state (33). The steroid binds to a site formed between αM1 and αM6 domains, plugging the ion pathway from the extracellular side. A high-affinity interaction is formed between the steroid and the αM1–2 part from domain α, which is rotated following the phosphorylation. Solving the crystal structure at 3.4 Å resolution of the phosphorylated pig kidney NKA in complex with ouabain has revealed that the steroid binds with intensive hydrogen bonding network to the αM1, αM2, and αM6 transmembrane segments (34). It was concluded that the binding pocket in the [Mg**^2+^**] E2P state allows deep ouabain binding with possible long-range interactions with Mg**^2+^** and K**^+^** ions.

Applying fluorescein-labeled ouabain and a lanthanide binding tag in the NKA derived from *Xenopus oocytes* the data of spectroscopic measurements (35) suggested two different type binding sites for ouabain: one external, low-affinity site on the extracellular end of the ion pathway as previously assumed; the second high-affinity site is slightly deeper toward the intracellular end of the ion pathway, as indicated by recent X-ray diffraction studies.

In conclusion, the high-resolution crystal structure data have identified the location of ouabain in the NKA together with the protein domains involved in the interaction with this CS. The ouabain-binding site described in Ref. (32) has a flat hydrophobic surface suitable for the interaction with the steroid frame as shown by Figure 1A.

For some hydrophilic ATPase ligands such as the decavanadate or the here disclosed spherical oligo-silicic acid (SOSA) (see below), it is improbable to target the hydrophobic receptor site of CSs and thus to compete directly with ouabain or digoxin. A more suitable target for hydrophilic inhibitors in P-type ATPases is the phosphorylation site in the cytoplasmic region between the N, A, and P domains (Figure 1B), as it was suggested for the decavanadate ion in SERCA (19).

The high-structural analogy between the hydrophilic poly-anionic decavanadate and the here disclosed SOSA suggests that SOSA should target ATPase pumps at the phosphorylation site in a similar manner as decavanadate. This assumption is illustrated by Figure 1B) with the high-resolution structure of the cytoplasmic domain of SERCA with the decavanadate ion bound to the hydrophilic phosphorylation site (19).

## Dilemma: EDLF or Cardiac Steroids

The physiological role of the CS receptors and the existence of endogenous non-steroidal ligands of NKA have been investigated by the group of Lingrel (36, 37). The experiments were conducted among others on some ouabain-resistant and ouabain-sensitive isoforms (cc2, cc3, and cc4) genetically engineered in mice and rat. The biological function and significance of the CS binding site was evidenced with rigorous distinction between cardiotonic steroids (CTS) and the yet undisclosed endogenous ATPase ligands. Reviewing the whole set of his experimental results and their significance for the physiological role of the NKA receptors, Lingrel concluded three main points: (1) the ouabain-binding site of the Na/K-ATPase plays a physiological role, (2) an endogenous ligand for the Na^+^/K^+^-ATPase must exist, and finally, (3) whether the endogenous ligand acts through a change in intracellular Na**^+^** or through a signaling mechanism is unknown (37).

Despite the convincing arguments of Lingrel, the relation between EDL factors and CSs remained a dilemma, i.e., “a problem offering two possibilities, neither of which is acceptable.” In fact, there are some experimentally identified but physically not isolated (probably labile) EDL factors assumed as ATPase ligands that differ markedly from CSs except for the inhibition of the NKA. Lacking weighable amounts of EDL factors, the studies have been performed only with the commercially available CSs and, therefore, could neither demonstrate nor exclude the identity of EDLF with CS.

This unsolved dilemma has marked the extensive research work focused to disclose the structure and properties of the putative endogenous ligands of the ATPases as thoroughly reviewed by Goto et al. (38), Hollenberg and Graves (39), Buckalew (40), Schoner and Scheiner-Bobis (41), Nesher et al. (42), and Bagrov et al. (43).

Numerous attempts to isolate pure EDL factors from various biological sources (organs, glands, plasma, or urine) were listed by these surveys (38–42). Despite applying extraction procedures on several kilogram amounts of starting material and efficient separation techniques, the final yields after multiple purification steps were invariably small: trace, sub-microgram amounts of EDLF, definitely insufficient for structural studies.

The low chemical stability of the EDL factors should also have been considered as possible explanation for the dramatically vanishing EDLF amounts along the purification processes. But this instability was not investigated in detail. Once a labile sodium pump inhibitor was signalized in peritoneal dialyzate (44) with even significantly higher inhibitory potency than ouabain, but the experiment was not reproduced. Of historical interest is the mention published 60 years ago by Szent-Györgyi (2) that, “the cardio-active serum factor if lyophilized and stored, loses its activity, as it also loses it on repeated freezing and thawing.”

Because of these persistent failures to isolate EDL factors, the search for endogenous ATPase ligands and assessment of their putative biological role became increasingly discrepant. Lacking measurable amounts of the pure EDLF on one side and the growing body of evidences that toxic CSs of herbal or amphibian origin are unable to function as endogenous ATPase ligands in mammals became an unsolvable problem. Further controversies were caused by the sugar moieties of the cardiac glycosides comprising desoxy-sugars (e.g., rhamnose, digitoxose) which were never identified in mammals, making it unlikely that such desoxy-glycosides can genuinely exist and act as endogenous ligands in these animals.

Estimated from their evolutionary history CSs identified in flowering plants could not be older than 100 million years and steroids from amphibians must be “younger” than 400 million years. It is very unlikely that such compounds have existed as regulatory ligands of the ATPase pumps 3500 million years ago in the prokaryote membrane.

After the successful evolution of the archaic ATPase 3.5 billion years ago, it was no more evolutionary pressure to improve this perfectly working ion-pumping mechanism or to change its endogenous regulatory ligand. The adaptation on the growing complexity of multicellular organisms was accomplished by diversification of the subunits or by the combination of substructures, without essential changes of the basic pumping mechanism.

Finally, it should be remarked that the search for identification of the EDL factors had considered almost exclusively organic candidates (38). This was not fully justified as the essential chemical reaction of the pumping cycle is the binding (and release) of a simple inorganic phosphate group. The lack of specific 3D structures in solution makes simple inorganic salts rather unable to fit a receptor site and to work as endogenous ligands contrary to organic substances. However, some inorganic poly-oxo-acids derived from metalloids such as Be, Al, Si, Ge, As, V, Cr, or Mn are able to form 3D structures in solution to fit into a receptor site. Actually the predominant part of these poly-oxo-acids and their salts are toxic for living organisms, which reduces the number of candidate endogenous ligands.

Several criteria of the putative EDLFs have been formulated previously, among others by Goto et al. (38). Completing the earlier list with a few criteria, we consider that the endogenous ATPase ligands should have characteristics as:

- inhibitor of the Na^+^/K^+^-ATPase,
- inhibitor of other P-type ATPases,
- ubiquitously distributed,
- bioavailable and eliminable,
- non-toxic for animals,
- was present in a very early stage of the evolution,
- sensitive to drying and freeze-drying,
- specific 3D structure in solution.

These properties can also explain the difficulties met earlier in the isolation and characterization of pure EDLFs. Further efforts are needed to disclose the detailed mechanism of action at cellular and physiological level of the actual EDL factors.

## ATPase Inhibitory Vanadates

The inhibition of the Na^+^/K^+^-ATPase by vanadate was discovered in year 1977 with the accidental observation (45, 46) that the reagent grade ATP of Sigma was contaminated with an ATPase inhibitory substance, identified as sodium vanadate (Na**_3_**VO**_4_**). The similar (isoelectronic) structure of the vanadate and phosphate ions was considered as a probable mechanism of the inhibition by vanadate in competition to the phosphate binding site.

Investigating the interaction of vanadates (47) with fluorescein-labeled SERCA, it was observed that vanadate impeded the high-affinity Ca^2+^ binding to the enzyme at 4°C. Vanadate inhibits the phosphorylation reaction by inorganic phosphate but had no effect on the phosphorylation by ATP. It was suggested that vanadate binds to the low-affinity ATP binding site of the ATPases, which is exposed only in the E2 conformation of the enzyme.

Interactions between SERCA and vanadate ions in solution have been investigated by **^51^**V-NMR spectra indicating that mono- and oligo-vanadates are bound to SR membrane influencing the structure of Ca^2+^-ATPase (48). Actually, the mono and oligo-vanadate species form some complex equilibria impeding the establishment of rigorous structure–activity correlations.

In the presence of Ca**^2+^**, it was observed that tetra- and deca- vanadate [V_10_O_28_]**^6-^** binds to the SERCA pump, whereas mono- meric vanadate binds to the SR only when ATP is present. There are further arguments that decavanadate clearly differs from mono- or small-vanadate oligomers in preventing the accumulation of Ca**^2+^** ions by SR vesicles, which is coupled to ATP hydrolysis (49).

Biological studies with vanadium often disregarded the formation of decameric vanadate species known to manifest high-affinity interaction with many proteins such as myosin and the SR calcium pump (50). Vanadium is accumulated in mitochondria in particular when decavanadate is administered. These findings point out the contributions of decavanadate to *in vivo* effects induced by vanadium in biological systems.

An increasing volume of data suggests the putative biological importance of decavanadate, a vanadate oligomer that eventually occurs in the cytoplasm more often than expected (51). Specific interactions of decavanadate have been clearly demonstrated for Ca^2+^-ATPase, myosin, and actin, considered as major proteins in muscle contraction and its regulation. Based on crystal structure data, the binding of the SERCA inhibitory decavanadate was localized (19, 52) to the ATP binding site between the cytoplasmic domains A, N, and P of the thapsigargin-inhibited enzyme in the absence of Ca^2+^ as shown by Figure 1B of the present paper.

Vanadate compounds show a significant antidiabetic efficacy. Sodium vanadate was applied in diabetes therapy 22 years before the first use of insulin to treat diabetes in human beings (53, 54). Besides its insulin-mimetic action, vanadate inhibits the glucose-6-phosphatase (G6P) enzyme, with a key role in glucose metabolism.

The proposal of Kramer et al. (55) to consider vanadium di-ascorbate with a molar mass of 403 Da as a candidate EDLF is worth mentioning. However, this hypothesis has not been confirmed since vanadate ions are not ubiquitously distributed in mammals and are toxic in particular by accumulation in some organs.

## ATPase Inhibitory MCS-Factors

Our way to disclose the structure of the assumed EDLFs is a typical example of serendipity. At the end of 1990s, we investigated at the Max-Planck Institute for Biochemistry in Munich an herbal product isolated from the roots of *Helleborus species*. The plant product contained, besides other components, the cardiac glycoside hellebrin with strong NKA-inhibitory potency. The chemical stability of hellebrin was monitored by measuring the inhibition of Na^+^/K^+^-ATPase, starting therewith a very intensive and prolific collaboration with Hans-Jürgen Apell and Robert Stimac from the University of Konstanz (GER), which led finally to the disclosure of the novel ATPase-inhibitory factor.

The alkaline treatment destroyed hellebrin and annihilated the NKA-inhibitory effect but, as the alkaline boiling was accidentally prolonged for several hours, a very potent novel NKA inhibitor was generated (56). By the HPLC on RP-18 column, the pure inhibitory compound eluted closely after the injection peak, or delayed if it was attached to some lipophilic components. Similar characteristics have been reported by the HPLC analysis of the earlier EDLF preparations from biological samples (38–43).

The main component of the plant material subjected to alkaline boiling was a resin-like compound, similar to the polymeric carbon suboxide (57). Therefore, it was thought that the obtained potent ATPase inhibitor could be a low molecular weight decomposition product of this polymer. For the structure of the de-polymerization product, we assumed a repeatedly condensed 4-pyrone frame that forms a supplementary cage-type macro-cycle with formula (C**_3_**O**_2_**)**_n_** where (n = 4, 6, or 8). We named the inhibitor MCS, macrocyclic carbon suboxide (56). Tentatively, this MCS was suggested as probable EDLF and natriuretic factor (58).

MCS factors showed a rigorously reproducible potent inhibition of the Na^+^/K^+^-ATPase, Ca^2+^-ATPase, H^+^/K^+^-ATPase, and K-dp-ATPase with IC_50_ values in the 0.2–0.5 μg/mL range. The mechanism of Na^+^/K^+^-ATPase inhibition by the MCS factor was investigated with the fluorescent styryl dye RH421, a dye known to reflect changes of local electric fields in the membrane dielectric. It was found that the binding of the MCS to the Na^+^/K^+^-ATPase is not competitive with ouabain (59, 60). MCS factors interact with the Na^+^/K^+^-ATPase in the E1 conformation of the ion pump and induce a structural rearrangement that causes a change of the equilibrium dissociation constant for one of the first 2 intracellular cation binding sites. The MCS-inhibited state was found to have bound one cation (H^+^, Na^+^, or K^+^) in one of two non Na^+^ specific binding sites, and the other Na^+^ ion was bound at high Na^+^ concentrations to the highly Na^+^-selective ion-binding site (60).

The proposal with cage-form condensed macrocyclic carbon suboxide structure was apparently supported by the mass ion peaks (m/z) containing multiples of the 68.03 Da unit, the molar mass of the C**_3_**O**_2_**.

**Figure 2:**
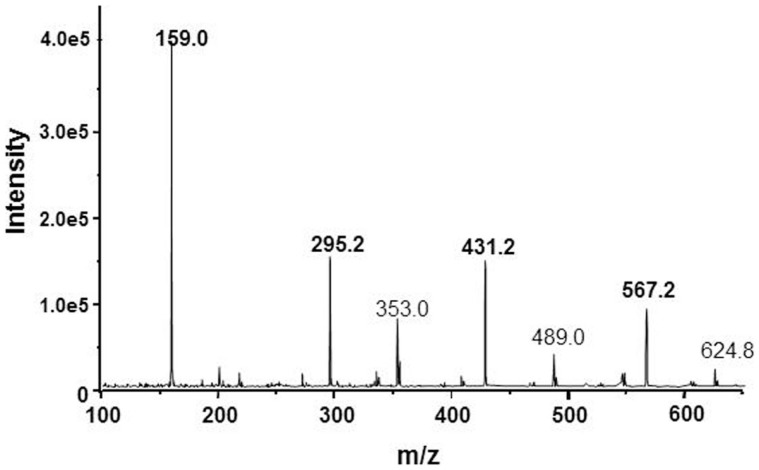
**ESI-mass spectrum of the NKA-inhibitory MCS factor corresponding to some mass ion fragments resulted by decomposition of SOSA**.

The main m/z peaks in ESI-MS spectra (Figure 2) have been assessed as small multiples of the carbon suboxide unit (68 Da) with 1 Na**^+^** ion according to the formula [(C_3_O_2_)**_n_**·Na]**^+^** thus, m/z = 159 Da correspond to *n* = 2; 295 Da (*n* = 4); 431 Da (*n* = 6), and 567 Da (*n* = 8). The small MH**^+^** peaks at 275, 409.2, and 544.2 Da were also perceptible in the mass spectrum (56).

Interestingly, a molar mass ion at M = 408 Da (MH**^+^** = 409; MNa**^+^ ** = 431 Da) was identified in some earlier EDLF preparations from biological sources, e.g., human plasma (61), placenta (62), or bound to a hypertension-associated plasma protein (63). It can be speculated about the possible identity of these factors and our MCS product but only the same molar mass ion value is not sufficient to prove or disprove this identity. The fine structure of the mass spectra may also differ due to the different ionization techniques, i.e., FAB used by Weiler et al. (63) and ESI-MS applied by us (56).

Although the ATPase-inhibitory effect of the MCS factors on several P-type ATPases and the mechanism of action on NKA were rigorously reproducible, the structure with head-to-tail condensed pyran-4-one rings supplementary bond in a cage-like macro-cycle could not be confirmed by synthesis despite huge experimental efforts with Frank Freudenmann and Luis Moroder at the MPI for biochemistry in Munich.

Likewise not confirmed were the specific **^13^**C-NMR signals and the UV-absorbance peaks expected for the macrocyclic condensed pyran-4-one structure. The assessment of the mass spectrum as multiples of a 68 Da unit was correct but the attribution of this mass to C**_3_**O**_2_** was erroneous. These disagreements required the revision of the proposed macrocyclic cage-structure.

## Spherical Oligo-Silicic Acid (SOSA)

The decisive hint to disclose the actual chemical structure of our NKA-inhibitory factor came from revision of the blind probe of the described alkaline preparation. Surprisingly, the several hours boiling of the NaOH solution alone, without any other reagent yielded a similarly potent ATPase inhibitor as that obtained by alkaline boiling of the plant polymer.

The mystery was explained by identifying small amounts of sodium silicate in the solution leached from the glass flask by its prolonged alkaline heating in oil bath at 120°C. Applying the proper neutralization-activation procedure (56), this silicate was transformed to the highly active ATPase-inhibitory factor identified as spherical oligomers of silicic acid. The generation of biologically active oligomeric condensation products from the inactive monosilicic acid was totally unexpected and thus very surprising.

We considered that this finding could have implications in clearing of some controversial disputes within the following research areas:

Regarding the biological role of silicon, it was generally agreed that Si provides structural support in plants and is beneficial of bones and elasticity of cartilages in animals. But neither a Si containing biologically active substance, nor a protein, which needs Si has been found in animals. Identifying the SOSA as biologically active water-soluble Si compound (64), a decisive argument has been provided in support of the assumed biological role (essentiality) of this element.

The assumed existence of EDLFs and their putative identity with cardiac glycosides was the subject of debates for several decades. The proposed identity of EDLF (65) with the SOSA was a novel approach suggesting the reconsideration of the divergent opinions.

The structure of the ATPase-inhibitory SOSA is formed by successive condensation of a few (oligos in Greek) molecules of monosilicic acid H**_4_**SiO**_4_** according to the equation (Eq. 1) where “*n*” should be in the range of 16–200.

(1) $$\begin{array}{l} \begin{array}{ccc} {\text{nH}}_{4}{\text{SiO}}_{4} & \to \; & {\left[\text{Si}{\text{O}}_{\text{x}}{\left(\text{OH}\right)}_{4-2\text{x}}\right]}_{\text{n}}\\ \text{mono-silicic acid} & & \text{oligo-silicic acid}\\ \text{x}=1.5 & \to \; & {\left[\text{Si}{\text{O}}_{1.5}\left(\text{OH}\right)\right]}_{\text{n}\;}\\ & & \;\text{SOSA} \end{array} \end{array}$$

**Figure 3:**
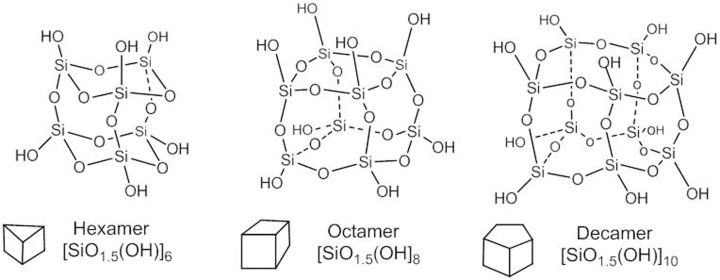
**Structure of polyhedral silsesquioxanes**.

The value *x* = 1.5 in the general formula [SiO**_x_**(OH)**_4-2x_**]**_n_** is congruent with a particular symmetry of the multi-cyclic silicic acid oligomers corresponding to polyhedral symmetry, i.e, prismatic hexamer (*n* = 6), cubic octamer (*n* = 8), and prismatic decamer (*n* = 10) structure, known as silsesquioxanes with general formula [SiO**_1.5_**OH]**_n_** and shown by Figure 3.

The spherical form of the oligo-silicic acid SOSA is accomplished for *x* = 1.5 in the general formula [SiO**_x_**(OH)**_4-2x_**]**_n_** and is assumed as the natural continuation of the polyhedral series with formula [SiO**_1.5_**(OH)]**_n_** for values of *n* > 20. There is an interesting formal resemblance with the series of Platonic bodies (tetrahedron, cube,…sphere) from geometry.

Actually, the cage-type condensed polyhedral silsesquioxanes have all Si-OH groups at the external vertices with nearly axial orientation. A similarly external distribution of the Si-OH groups is accomplished by the here disclosed nearly spherical oligomers of the silicic acid. SOSA molecules as next term in the series of polyhedral structures with the same general formula [SiO_1.5_(OH)]_n_ have the same ratio between the Si: O: H atom = 1: 2.5: 1.

But, there are significant differences between the chemical structure and properties of the polyhedral and of the spherical silica. The predominant difference is that polyhedral silsesquioxanes inhibit neither Na^+^/K^+^-ATPase nor other ATPases conversely to the strong ATPase-inhibitory SOSA. Structurally, all polyhedral silsesquioxanes (Figure 3) comprise only Q3 type Si atoms, i.e., each one is involved in three (Si)-O-Si bonds and one (Si)-OH bond.

**Figure 4:**
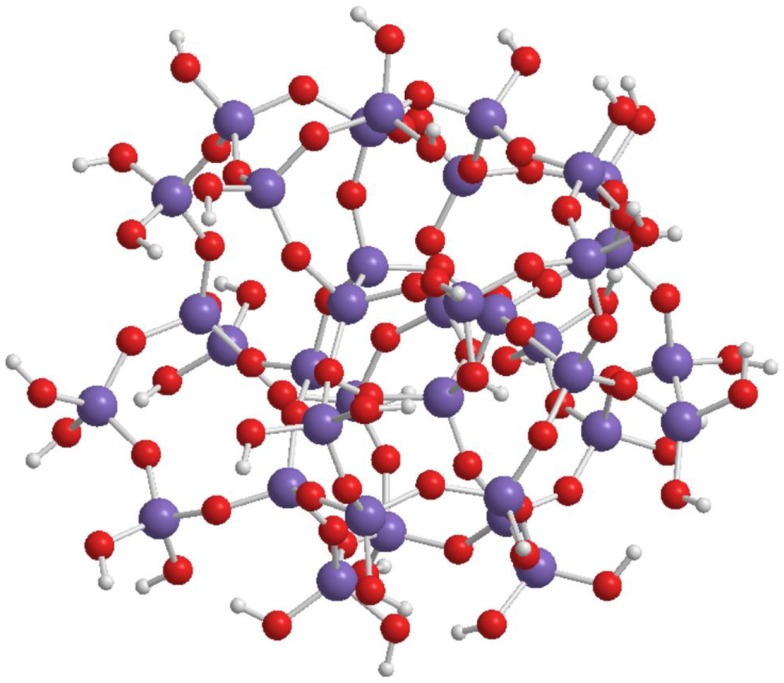
**Structure of SOSA with formula: [Si_36_O_54_ (OH)_36_] with 36 Si-OH bonds on the external surface of the spherical molecule**.

The spherical shape of the oligo-condensed silicic acid with *x* = 1.5 is accomplished by some preferred “*n*” values. Figure 4 shows the SOSA molecule with *n* = 36, as ball and stick model. This SOSA molecule with formula [Si**_36_**O**_54_** (OH)**_36_**] comprises in its internal shells 4 + 8 = 12 Si atoms of type Q**^4^** (without Si-OH bonds). In the external shell, there are 12 Si atoms of type Q3 (with one Si-OH bond) and 12 Si atoms of type Q**^2^** (with two Si-OH bonds). It is observed that the 36 external Si-OH bonds are displayed on the external surface strongly facilitating the hydrophilic interactions with proteins.

Applying the gel-permeation chromatography (GPC) method, molar mass values in the range of 1.2–6.0 kDa have been obtained of the spherical condensed silicic acid oligomers. For the SOSA molecule illustrated on Figure 4 there resulted *M* = 3.2 kDa, corresponding to molecular diameter ϕ = 2.2 nm further confirmed by dynamic light scattering (DLS).

The mass ion peaks of SOSA obtained by ESI-MS technique were practically the same as those identified in the spectrum of our MCS factor (Figure 2). Actually these m/z peaks correspond to small ionic fragments resulted by the split of the large SOSA molecules in the ionization chamber. The main mass ion peaks correspond to the formula [(Si**_2_**O**_5_**)**_q_**·Na]**^+^** with m/z = 159 Da for *q* = 1; 295 Da for *q* = 2; 431 Da for *q* = 3, and 567 Da for *q* = 4. Similar mass spectra have been observed by decomposition of condensed silsesquioxanes (66, 67).

In fact, the erroneous assessment of the mass ion peaks as derived from carbon suboxide was caused by the accidental identity of the molar mass of C_3_O_2_ M = 68.03 Da and the mass of the 1/2 Si**_2_**O**_5_** (di-silicate) ion with M = 68.08 Da, resulted by the decomposition of SOSA. In conclusion, the mass ion peaks on Figure 2 correspond rather to the formula [(Si**_2_**O**_5_**)**_q_**·Na]**^+^** of SOSA and not to the formula [(C_3_O_2_)**_n_**·Na]**^+^** as initially suggested.

## SOSA as Possible Endogenous Factor

With the confirmed potent inhibition of several P-type ATPases, SOSA fulfills the main condition as a candidate ATPase ligand. Its classification as an endogenous factor requires further that SOSA should be produced within the organism, tissue, or cell. This condition is satisfied with the presumed biosynthetic pathway of SOSA by spherical oligomerization of the monosilicic acid (H_4_SiO_4_) ubiquitously distributed in plants and animals (68, 69).

A human body contains approximately 1 g of Si in various combinations with oxygen (generic name: silica). Almost all silica in the body is bound to biomolecules and tissues and only a minor part circulates as dissolved silicic acid in blood plasma and urine (70). The plasma level of silicic acid is 0.7 mg/L corresponding to 0.2 mg/L silicon. Infants have two- to three-fold higher Si plasma level, but in contrast aged persons and pregnant women have significantly lower values of Si. For human beings, the daily ingested amount of Si is estimated to be 30–50 mg and the same amount is eliminated in urine. After meals, the plasma concentration of Si increases 30–50%, and returns after a few hours to the initial level (71). The regulatory mechanisms of Silicon homeostasis require elucidation.

The catalytic action of a biomolecule to accomplish the spherical oligomerization of silicic acid yielding SOSA is presumed. Proteins catalyzing high-grade polymerization of silicic acid have been found (67) in algae (silaffin) or sponges (silicatein). Biomolecules favoring the spherical oligomerization of silicic acid have not yet been identified.

The mechanism of ATPase regulation by the probable endogenous ligand SOSA is not fully understood. According to the unusual physical–chemical properties of SOSA and to its specific interactions with the Na^+^, K^+^, H^+^, Mg^2+^, or Ca^2+^ ions and with the ATPase protein, some challenging proposals for the regulation mechanism may be formulated.

### Assumed Regulatory Mechanism of SOSA

Transmembrane ion pumping may be physically influenced by SOSA located in a receptor cavity along the ion-transport pathway of the ATPase molecule. The SOSA molecule should behave like a multi-anionic gel with selective binding or permeability effects on cationic species depending on their concentration, charge and size.

The interaction of SOSA with the ATPase protein is assumed on the cytoplasmic site of the ATPases in the E1 conformation as revealed by charge transfer investigations with the dye RH421 (60). According to these data, SOSA should inhibit the X1E1 state, manifesting complex interactions with different X ions (Na^+^, K^+^, or H^+^) and with Na^+^ ions in the (Na)NaE1 state. Structural details of the very complex binding of the sodium ions to the NKA in the state preceding phosphorylation have been disclosed by high-resolution crystal structure (72).

The ion-sensitive nature of our ATPase inhibitory factors MCS (disclosed as SOSA) was remarked in Ref. (60) assuming that the concentration of the Na^+^ and of other ions may cause significant structural rearrangements. The *in vitro* ion-sensitive structure of SOSA is also supported by DLS measurements. Tentatively, it could be assumed that the SOSA structure at [Na^+^] < 5mM inhibits the sodium pump but the structure at [Na^+^] ≥ 5 mM should activate it. A possible activation mechanism could be by favoring the differentiate binding of Na^+^ ions to the E1 conformation and promoting the phosphorylation step. Accepting this hypothesis, the generally found cytosolic level of [Na^+^] = 5.0 mM in the eukaryotic cells could be a consequence of the structural change of the archaic ligand SOSA, which happens accidentally at this Na^+^ ion concentration.

The here proposed probably archaic regulatory mechanism of the NKA pump by ion-dependent structural changes of the endogenous ligand could also work for other ATPases. The ion concentration threshold for the activation of other ATPases should depend upon the concentration of the ions to be pumped and their interaction with SOSA. This regulatory mechanism, assuming the ion-sensitive structural variation of the archaic ligand SOSA, can explain the astonishing manifoldness of the same well-conserved ion-pumping mechanism in the hitherto identified more than 200 different P-type ATPase pumps.

### Conclusions and Outlook

With almost all Si-OH bonds disposed on the external surface of SOSA, this substance should bind preferentially to hydrophilic domains of the target proteins. In P-type ATPases, the well-conserved phosphorylation site between the cytoplasmic domains P and N provides an adequate binding site for SOSA molecules similar to decavanadate (Figure 1).

Although SOSA inhibits NKA at sub-micro-molar concentration, its direct competition with ouabain for the hydrophobic steroid binding site in NKA is less probable. The non-competitive binding mechanism of our hydrophilic ATPase inhibitor and ouabain was confirmed by the fluorescence dye measurements of Stimac et al. (59, 60). An apparent competition may appear if the NKA conformation required for SOSA binding and that required for ouabain binding are different (C. Toyoshima, personal communication).

The ion-concentration dependent structural changes of SOSA suggest a probable archaic regulation mechanism of the sodium pump and of other ATPases where the pump ligand is sensing the nature and molarity of the ions to be pumped. It is a challenging idea that the transmembrane ion pumping with fundamental importance for many essential life processes should be regulated by the cation-sensitive structure of an inorganic acid.

The identified chemical properties and enzymatic activities of SOSA are congruent with the predicted characteristics of the EDLFs of the P-type ATPases. Table 1 shows a synoptic presentation of the assumed characteristics of the putative EDLF factors in comparison with that of candidate substances: ouabain, marinobufagenin, vanadate, and the here disclosed SOSA.

**Table 1:** **Comparison of the endogenous ATPase ligand candidates**.

		EDLF	Ouabain	Marino-bufagin	Vanadate	SOSA
**Characteristics**						
C-1	Chemical nature	ND	Cardiac glycoside	Bufadienolide steroid	Inorganic, poly-oxo-acid	Inorganic poly-oxo-acid
C-2	Molar mass (kDa)	0.4–5.0	0.58	0.4	0.12–1.5	1.4–6.0
C-3	Stability by drying	Low	Stable	Stable	Limited	Low
C-4	Structural stability	ND	Stable	Stable	pH sensitive equilibra	Cation and pH sensitive
C-5	Nature to ATPase binding site	ND	Hydrophobic	Hydrophobic	Hydrophilic	Hydrophilic
C-6	Distribution	Ubiquitous	Only in a few plant	Predominantly in	Limited	Ubiquitous
			species	amphibians
C-7	Mammalian occurrence	Yes	No	No	Only in traces	Ubiquitous
C-8	Evolutionary age million years Myr	>3500 Myr	<60 Myr	<360 Myr	>3500 Myr	>3500 Myr
**Biochemistry**						
B-1	Toxicity	Low	High	High	Moderate	Low
B-2	Biosynthesis	Predicted	Only in a few plant	Predominantly in	Improbable	From monosilicic-acid
			species	amphibians
B-3	Na,K-ATPase	Inhibitor	Inhibitor	Inhibitor	Inhibitor	Inhibitor
B-4	SERCA	Inhibitor	No	No	Inhibitor	Inhibitor
B-5	H/K-ATPase	Inhibitor	No	No	Inhibitor	Inhibitor
B-6	K-db-ATPase	Inhibitor	No	No	Inhibitor	Inhibitor

*ND: not determined*.

*Summarizing* the characteristics of the SOSA, it may be concluded that these match the predicted criteria of the endogenous ligands of the NKA and probably of further P-type ATPases:

- SOSA inhibits with similar potencies (IC_50_ ~ 0.2–0.5 μg/mL) the ouabain-sensitive Na^+^/K^+^-ATPase from rabbit medulla and the ouabain-insensitive enzyme from rat.
- SOSA inhibits Ca^2+^-ATPase from SR, H^+^/K^+^-ATPase from gastric membrane and of K-dp-ATPase from *Escherichia coli* with IC_50_ values in the range of 0.2–0.5 μg/mL.
- There are no sensitive methods to differentiate between mono and oligo-silicic acids in cells. But the assay of the NKA-inhibitory factors EDLF in urine and plasma suggest the probable presence of SOSA in these biological fluids.
- Monosilicic acid is present in almost all cells; thus, its adequate transformation into SOSA can occur *in situ*, catalyzed and/or controlled by proteins.
- Preliminary data revealed a reduced toxicity by per-oral and a moderate toxicity by intravenous or intramuscular administration of SOSA. Renal elimination is assumed.
- There are no reasons to doubt the presence of silicic acid and of SOSA in the early history of the evolution.
- SOSA is stable in solution for several years but it loses its activity by freeze-drying probably through the forced intermolecular condensation of water molecules.

It is planned to obtain further structural details of the interaction of SOSA with ATPase proteins with possible implication for the regulation of the pump. One of the very intriguing questions is to investigate the influence of the concentration of Na^+^, K^+^, Mg^2+^,Ca^2+^, and other ions on the structure of SOSA. The further great challenge for ongoing research is to establish the role of SOSA in ATPase related cellular and physiological processes and to explore its possible health-care applications in some connected pathologies (73, 74).

## Conflict of Interest Statement

Dr. Franz Kerek is founder and majority shareholder of the company SiNatur GmbH in Munich, Germany where the R&D activities of the novel factor SOSA were conducted. Prof. Victor A. Voicu has no conflict of interests.
